# The efficiency of different search strategies in estimating parsimony jackknife, bootstrap, and Bremer support

**DOI:** 10.1186/1471-2148-5-58

**Published:** 2005-10-29

**Authors:** Kai F Müller

**Affiliations:** 1 Nees-Institut für Biodiversität der Pflanzen, Rheinische Friedrich-Wilhelms-Universität Bonn, Meckenheimer Allee 170, Bonn, D-53115, Germany

## Abstract

**Background:**

For parsimony analyses, the most common way to estimate confidence is by resampling plans (nonparametric bootstrap, jackknife), and Bremer support (Decay indices). The recent literature reveals that parameter settings that are quite commonly employed are not those that are recommended by theoretical considerations and by previous empirical studies. The optimal search strategy to be applied during resampling was previously addressed solely via standard search strategies available in PAUP*. The question of a compromise between search extensiveness and improved support accuracy for Bremer support received even less attention. A set of experiments was conducted on different datasets to find an empirical cut-off point at which increased search extensiveness does not significantly change Bremer support and jackknife or bootstrap proportions any more.

**Results:**

For the number of replicates needed for accurate estimates of support in resampling plans, a diagram is provided that helps to address the question whether apparently different support values really differ significantly. It is shown that the use of random addition cycles and parsimony ratchet iterations during bootstrapping does not translate into higher support, nor does any extension of the search extensiveness beyond the rather moderate effort of TBR (tree bisection and reconnection branch swapping) plus saving one tree per replicate. Instead, in case of very large matrices, saving more than one shortest tree per iteration and using a strict consensus tree of these yields decreased support compared to saving only one tree. This can be interpreted as a small risk of overestimating support but should be more than compensated by other factors that counteract an enhanced type I error. With regard to Bremer support, a rule of thumb can be derived stating that not much is gained relative to the surplus computational effort when searches are extended beyond 20 ratchet iterations per constrained node, at least not for datasets that fall within the size range found in the current literature.

**Conclusion:**

In view of these results, calculating bootstrap or jackknife proportions with narrow confidence intervals even for very large datasets can be achieved with less expense than often thought. In particular, iterated bootstrap methods that aim at reducing statistical bias inherent to these proportions are more feasible when the individual bootstrap searches require less time.

## Background

"Without some assessment of reliability, a phylogeny has limited value" (Sanderson 1995: 299) – an examination of the recent phylogenetic literature shows there is a general agreement on this fact, and only few molecular phylogenetic studies of the past 15 years exist in which no estimate of confidence is provided. In the context of cladistic (parsimony) analyses, two basic types are most common: resampling plans (bootstrap, jackknife), and those based on the length difference of trees (Bremer support).

### Bootstrap and jackknife in parsimony analyses

Bootstrap and jackknife are computer intensive statistical methods for error estimation [1,2]. With regard to their applicability in phylogenetics [3,4], elaborate discussions exist in the literature [5-9]. The fact that fundamental statistical assumptions may not be met in the phylogenetic context (such as independent, identically distributed variables) could not prevent the bootstrap from becoming the most popular method for reliability assessment, in particular since many researchers consider bootstrap and jackknife merely as indications of relative support, not in a hypothesis-testing framework. Felsenstein [10] provides an easy-to-read but detailed description of resampling plans in phylogenetics and addresses solutions to circumvent some of these more fundamental problems.

Aside from that, the majority of applied phylogenetic studies hitherto do not provide a justification for using a certain number of replicates or a particular search strategy during each bootstrap (or jackknife) replicate. Unfortunately, often the parameter settings that are employed are not those that appear recommendable in view of the existing theoretical and empirical work that provides a guideline for the number of replicates [e.g., [11]] or search strategies [12-14] to be used. Phylogenetic trees are tools for understanding biological processes and gain more and more importance far outside the field of pure phylogenetics. Ideally, biological conclusions based on a given node in the tree should take into account the level of confidence one can have in the existence of the node. Therefore, it appears necessary to more efficiently spread the existing knowledge on the performance and interpretability of the bootstrap and jackknife under different circumstances, but also to address those questions that still remain open.

### The number of replicates

Hedges argued that at least 1825 replicates are needed if one wants to attain ±1% accuracy for bootstrap proportions of 95% or higher [11]. The underlying considerations are based on the binomial distribution, which has the favorable characteristic of a variance *σ*^2 ^that equals *np*(1-*p*). In the context of resampling plans, *n *is the number of replicates and *p *is the bootstrap or jackknife support value (bootstrap percentage or bootstrap *p *value) expressed as a fraction of 1, i.e., the proportion of replicates that yielded a tree containing a particular phylogenetic group. Therefore, the *n *needed to attain ±*a *accuracy (as 95% confidence interval spanning ±1.96 *σ*) at a support level *p *can readily be calculated as

*n *= *p *(1-*p*)(1.95996.../ *a*)^2^.     (1)

Hedges (1992) based the determination of the confidence interval upon an approximation of the binomial distribution by the normal distribution. However, when *np *≤ 5 and *n(1-p) *≤ 5 (roughly), much precision is gained when standard errors are based on the binomial distribution. This condition is easily fulfilled at high probabilities. Here, the confidence intervals become asymmetric.

Let *Y *be the number of successes (replicates that yield a given node) out of *n *trials (replicates), and *p *the success probability of each trial. The lower endpoint of the (1 - *α*)100%-confidence interval around the estimated *p *(derived from observing *m *= *pn *successes) is given by the *p*_*l *_such that



Similarly, the upper endpoint is defined by finding the *p*_*u *_such that





**Figure 1:**
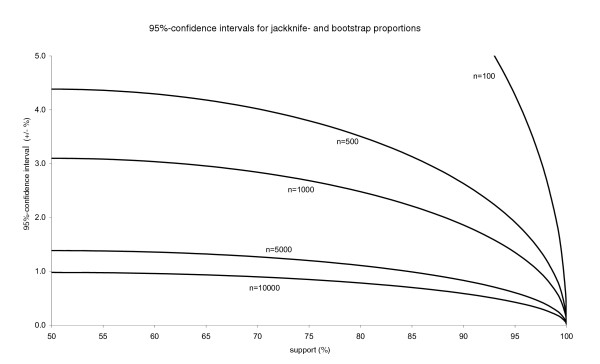
**95% confidence intervals at jackknife and bootstrap frequencies between 50 and 100**. 95% confidence intervals at jackknife and bootstrap frequencies between 50 and 100. For a detailed explanation see text.

Rather than odd numbers such as "1825", researchers currently use "500", "1000", etc. replicates, and the question arises, what the confidence interval for a given *p *may be when these number of replicates are used. This is easily derived from Eq. (1) or (more precisely) from Eqs. (2)-(3), and Fig. 1 graphs these intervals for support levels of ≥50 and common replicate numbers. With help of this graph, the frequent issue whether two apparently different support values really differ can relatively easily be addressed visually. The decrease of BS/JS (bootstrap or jackknife support) standard deviations with an increase of the number of replicates (Fig. 1), which theoretically follows from the above equations, was confirmed in detailed empirical studies on the topic using real datasets of Saxifragaceae [12] and Orchidaceae [15].

### Search extensiveness

From a theoretical point of view, the question of the optimal number of bootstrap replicates is easier to solve than that of the optimal heuristic search strategy to be applied during bootstrap replicates of cladistic analyses. Farris et al. [4] initially argued that thorough swapping during replicates is unnecessary. The whole point of using resampling is "to avoid drawing poorly-supported conclusions" [4: 117] and identifying those groups that are strongly supported by the data. The most strongly supported groups are certainly easily identified via bootstrapping or jackknifing when no swapping is performed, but at the expense of risking that some nodes are ignored that could gain significant support (whatever one regards as significant). 1 - *s *is conventionally interpreted as type I error (*α*) in hypothesis tests where a group of taxa will be considered monophyletic if BS > *s *(H_0_: group is not monophyletic, H_1_: group is monophyletic; *α*: probability of rejecting H_0 _while it is true; [16]). Thus, BS/JS that is systematically too low at least does not entail an increased danger of mistakenly inferring monophyly. It does, however, lead to rejecting monophyly incorrectly too often, which is not desirable, either. Nonetheless, one of Farris & al.'s main points was to contrast the speed of their jackknifing approach with the slower neighbor-joining bootstrap and extensive heuristic parsimony searches to identify MP trees (most parsimonious trees); branch swapping during each replicate would have strongly lowered the performance contrast. Therefore, the first version of Farris' jackknifing application, 'JAC', did not perform branch swapping at all.

A number of studies provided practical evidence from real datasets that the non-branch swapping approaches (as in JAC or the "fast" option in PAUP*) yield significantly lower support estimates than analyses performing some kind of branch swapping [12-14,17,18]. Accordingly, branch swapping was later added in the upgrade 'XAC'. In line with Farris & al.'s basic assumptions, however, it was concluded that swapping on more than 1 to 2 trees per iteration does not change support significantly [15]. This implied that the increased computational effort connected with more extensive searches per replicate does not necessarily translate into more accurate estimates. Note that these examinations used the random addition search strategy available in PAUP* [19], which is known to relatively soon fail to find shortest trees as the number of terminal sampled increases (Nixon, 1999). This is why cladistic analyses of datasets approaching or exceeding 100 to 150 taxa (although strongly depending on the dataset) usually make use of the parsimony ratchet [20] or other fast cladistic algorithms, available through a number of software tools [21-24].

These results raise the question in how far bootstrap percentages are affected by trees found in each bootstrap replicate being far from most parsimonious. The above-cited increase in search exhaustiveness, namely moving from non-branch swapping via NNI and SPR to TBR, yielded a considerable increase in support [12,18]. Swapping on more trees (via RAS) apparently did not increase, but sometimes even lessened the average support [15]. These investigations, however, were conducted on datasets for which it was quite likely that shortest trees could be encountered without much search effort per iteration because size and homoplasy still allowed that shortest trees could be found with intermediate effort without algorithms designed for particularly large datasets.

If the standard error of the BS/JS value itself is neglected (e.g., assuming bootstrap/jackknife searches with an infinite number of replicates and a confidence interval for each BS/JS frequency approaching zero width), a dataset-dependent graph can be imagined in which the bootstrap is a function of the exhaustiveness of the search. If we further ignore for a moment the effect of how many trees per replicate are actually used in the majority rule consensus [14], and whether tree weighting is employed for these, this graph will asymptotically approach a "BS/JS level of saturation" at which further increase in exhaustiveness does not increase BS/JS. As a rough guideline we expect that the more taxa, the later this level of saturation will be reached. Certainly other factors such as homoplasy and phylogenetic signal inherent to characters play a significant role, but the mere fact that the number of possible tree topologies soon reaches astronomical dimensions [25] provides the most severe limitations to search algorithms. Somehow a point has now to be chosen at which one decides that the increase in support is not worth the additional search effort. A statistic could be chosen that describes the support level for each discrete level of exhaustiveness and subjected to hierarchical significance tests.

Without the unrealistic assumption of an infinitely narrow confidence interval around each support level, things become more complicated. The size of the (let's say 95%) confidence interval depends on the support level one looks at, less dramatically so when the number of replicates becomes very large (Fig. 1). Thus, to a considerable extend, drawing conclusions on the relative merits of certain search strategies has to take into account the support levels of interest. Commonly, these will fall in the interval [80;100], but less likely only in the standard interval [95;100] due to the frequently cited conservativeness of the nonparametric bootstrap [14,16,26,27].

The relationship between the number of trees per replicate fed into the consensus calculation and the BS/JS is still less straightforward. The more conservative approach of using strict consensus trees of each replicate for the final consensus tree [e.g., [28]], referred to as "strict-consensus approach" (SC) by Davis et al. [29], can be expected to always result in equal or lower support than the standard approach in PAUP*, for which the term "frequency-within-replicates approach" (FWR) has been coined [29,30]. The latter employs tree weights that maintain information on nodes that other trees found in the same replicate lack. This theoretical expectation was empirically corroborated very recently using a 218-terminal dataset [29]. The SC approach [28] may more closely reflect the use of the bootstrap outside the field of phylogenetics, but is only rarely pursued in published analyses [e.g., [31-33]]. Restricting the discussion to the tree-weighting approach in PAUP*, it is hard to predict whether additional trees saved per replicate will decrease resolution of the final majority rule consensus, because these additional trees are usually also swapped upon and thus enhance the probability of finding trees closer to the optimal score of the current replicate. The latter effect is counter to the first, and which effect will be stronger depends on a whole array of parameters, probably above all on the sampling size and thoroughness of the search. Using a 173 taxa data set with 1180 parsimony informative characters, Freudenstein et al. found that beyond 2 trees, saving more trees considerably decreases overall support, if these trees are from the same RAS (random addition search) iteration and not obtained via additional RAS replications [15: 151]. The authors, however, did not test the effect of increasing search extensiveness beyond 2 RAS replicates per jackknife replicate.

### Jackknifing versus bootstrapping

Principally, all considerations below apply equally to the bootstrap and jackknife. Farris et al. argue that, in order to directly compare jackknife frequencies with bootstrap frequencies, the probability that a character appears in the resampled matrix has to be set to 1-1/e [4] (along with other requirements). This was recently emphasized again by Freudenstein et al. [15]. Felsenstein disagrees with that view, demonstrating that at least sometimes a 50%-deletion-jackknife more closely reflects bootstrap proportions [10]. In any case, when comparing the behavior of bootstrap and jackknife, differences in the support levels are also a function of the probability that a character appears in the resampled matrix and, thus, the amount of data used for tree inference in the pseudoreplicates.

### The Bremer support

The Bremer support [BrS, [34-36]], a synonym of "decay index" [37], "length difference" [38], or "support index" [SI, [39,40]], is a completely different measure of branch support and has been addressed in detail recently [41,42]. For intermediate to large datasets, the calculation of BrS can be problematic, because support values can turn out to be severe overestimations of support [35] unless relatively thorough search strategies are invoked to assess support of each branch. It has been demonstrated that using the parsimony ratchet during Bremer support analysis is highly advantageous in such cases [43]. However, the data-dependent optimal compromise between ratchet search time and improved support accuracy has not been addressed so far and therefore will be briefly dealt with below.

## Results and discussion

### Bootstrapping and jackknifing

Empirical studies on four different molecular datasets (see Methods) of 86, 89, 385, and 567 taxa, respectively, yielded the following results. Using 10 or 20 parsimony ratchet cycles per jackknife replicate instead of one simple addition search with one saved tree yielded no enhanced support for the 86 and 89 taxa datasets (sign test: p < 0.05). This confirms conclusions from RAS searches on data sets of similar dimension [100 taxa, ref. [13]] or twice as big [173 taxa, ref. [15]]. For the considerably larger 385 and 567 taxa datasets, 10 ratchet cycles could not enhance support significantly, either (sign test: p < 0.05). Moreover, the effect of saving more than one shortest tree per jackknife iteration and using a strict consensus tree of these for the final jackknife consensus tree became obvious. This is detailed in Table 1 for the 567 taxa tree: computing a strict consensus of the *n *shortest trees found during 10 ratchet iterations (0 <*n *< 11) provides less support than saving only one out of these shortest trees (p << 0.05). The same effect was previously observed in a 173 taxa dataset [15] when 20 trees were saved (in one RAS cycle per jackknife replicate, applying tree weights rather than using consensus trees): support decreased compared to the outcome of the same analysis saving only one tree (32 clades received at least 4% lower support; the others remained the same. This amounts to a highly significant effect given the number of replicates used by the authors). Consequently, for larger data matrices, reduced search effort (but not as much reduced as using no or less efficient branch swapping) yields rather slightly overestimated support compared to the support found with higher effort. While it has been shown that not applying TBR can severely underestimate support [12,15], using still more thorough search approaches (e.g., several RAS or ratchet cycles, saving several trees) does not significantly raise or even lowers support.

**Table 1:** Contrasting jackknife support at nodes of the 3-gene jackknife tree for three different search approaches. Contrasting jackknife support (from 500 replicates) at 429 resolved nodes of the 3-gene jackknife-50%-majority-rule-consensus tree (567 terminals) for three different search approaches per jackknife replicate. (1) one heuristic search saving one tree and using simple addition ("simple"); (2) 10 parsimony ratchet iterations, starting from a tree found with simple addition, using the first shortest tree only found within the 10 iterations for consensus tree calculation ("10sv1"); (3) as before, using a strict consensus of all shortest trees found within the 10 ratchet iterations per jackknife replication ("10svAll").

**Compared search strategy**	**Sign test, % first < second**	**Sign test, *p***	**Wilcoxon test, *T***	**Wilcoxon test, *p***	**Higher support in...**
Simple *vs*. 10sv1	54.9	0.173	10685.5	0.311	---
Simple *vs*. 10svAll	42.3	0.038	7442.5	0.005	Simple
10sv1 *vs*. 10svAll	6.9	0.000	1107	0.000	10sv1

In all, extending the search extensiveness beyond the rather moderate effort of TBR and saving one tree per replicate does not translate into significantly increased support. Compared to Bremer support, theoretical considerations show that there is a lower risk of significantly overestimating support by using less thorough searches (enhanced type I error of accepting a clade that in fact is not there). The lack of inflated estimates caused by less extensive searches was corroborated using real datasets [12] and simulation studies [13]. These studies, however, compared searches without branch swapping or less effective swapping with searches that include TBR, while not addressing the factor of the number of trees saved and used in the bootstrap/jackknife consensus. The investigation of Freudenstein et al. [15] with 20 saved trees already indicated that support might well drop with more conflicting topologies taken into account per jackknife replicate. In their experiment, however, the option of saving (and swapping on) 20 trees also enhanced the likelihood of finding still shorter trees per replicate. In the present study, the pure effect of ignoring all but one of the topologies with the best score per jackknife replicate becomes evident: support is significantly higher than in a jackknife consensus tree based on all shortest trees found. Thus, for very large trees, there appears to be a small risk of overestimating support. However, this risk probably cannot be judged problematic, in particular not in view of the general conservativeness of nonparametric jackknife and bootstrap estimates in phylogenies [14,16,26,27], which in general should more than counterbalance this slight effect; at least it does so for the dataset analyzed here. As a response to this general conservativeness, *α*-levels have been raised far above the common 1% – 10% in empirical phylogenetic studies, leading to the acceptance of the presence of nodes with <<90% BS, or at least to referring to such nodes as "highly supported" [e.g., [44]]. One could easily recommend reducing this small risk of overestimating support by representing each bootstrap replicate by a consensus tree derived from multiple searches, but this probably suffers from a too high cost-benefit ratio to be practical in most analyses: 10 ratchet iterations require roughly a 20-fold search time compared to a simple search.

On the other hand, even for large trees, there appears to be no severe risk to underestimate support, as long as one simple-addition tree is swapped using TBR. In contrast, using random addition or random trees as starting trees during each replicate leads to highly significantly underestimated support (tested for the 385 taxa dataset; sign test: *p *<< 0.05).

Note that the above considerations aim at contrasting search strategies and do not extend to fundamental statistical bias existing in bootstrap and jackknife proportions [5,6,45,46] that may deteriorate with increased taxon sampling [14] but improves with increased character sampling. For datasets with many taxa, computational limitations of search strategies become confounded with this bias. Therefore, Sanderson and Wojciechowski [14] could not preclude that part of the decline in BS they observed when increasing sampling size (≤140 taxa) was due to the failure of the simple addition search (saving one tree) to find shortest trees. In view of the results presented here, this effect was probably negligible compared to the statistical bias from random homoplasy distributed among taxa [explanation 3 in 14]. To reduce this bias and achieve more accurate confidence limits, much more computer-intensive, iterated bootstrap methods have to be taken [e.g., [5,45-47]], frequently thought to be too time-consuming to be practical for large amounts of data. Sanderson and Wojciechowski argue that relying on search algorithms with only little branch swapping may circumvent this computational limitation, allowing multiple rounds of bootstrapping by saving time during each individual bootstrap [14: 684]. The outcome, they say, may still be somewhat too conservative because of the failure of these algorithms to find MP trees, but would still be more indicative of true support than conventional BS/JS. In light of the performance of varyingly extensive search strategies on datasets even larger than that in ref. [14], it appears that iterated bootstrap methods are not as impractical as previously thought and should more frequently be considered.

Finally, as shown in Fig. 1, the higher the number of replicates, the lower the error margin for the BS/JS support. To evaluate differences in support for a particular clade, confidence intervals have to be kept in mind to arrive at a statement on the significance of differences. The number of replicates needed to narrow down the confidence intervals to a desired level is a function of the BS/JS. If, for example, one restricts conclusions from a tree topology to nodes >90 and is happy with knowing (at a 5% risk) that a "91" cannot equally likely be a "89" (±1%), more replicates than 3458 are not needed.

### Bremer support

**Figure 2:**
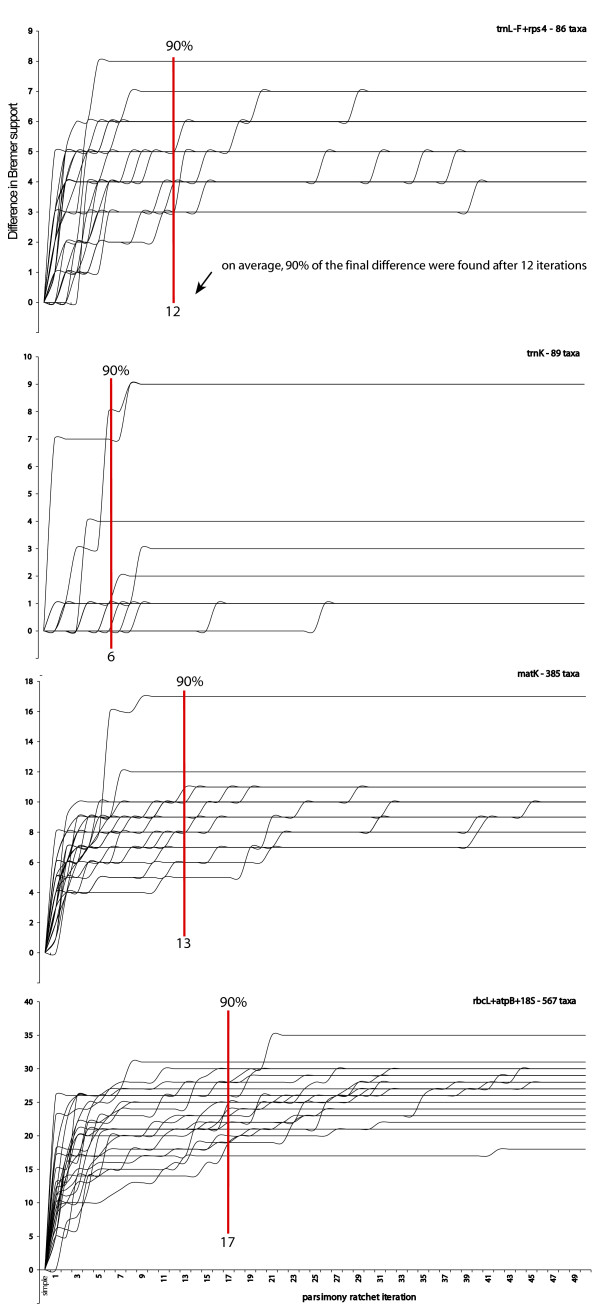
**Differences in Bremer support for 25 randomly selected nodes**. Differences in Bremer support for 25 randomly selected nodes (x-axis), comparing values obtained by a simple search (saving 1 tree) with those obtained with subsequent parsimony ratchet iterations (y-axis). Vertical lines mark the average number of iterations at which 90% of the final support difference (observed after 50 iterations) are exceeded first.

Fig. 2 shows how the Bremer support develops at the 25 randomly selected nodes with increasing search extensiveness. Values obtained by a simple search with TBR branch swapping (saving 1 tree) are compared with those obtained with 1 – 50 parsimony ratchet iterations. Vertical lines mark the average number of iterations at which 90% of the final support difference (observed after 50 iterations) are exceeded first. In the smallest dataset (86 taxa) this is the case at the 12^th ^ratchet iteration. For the slightly larger 89 taxa dataset, less iterations are needed until support values become comparably saturated. This dataset, however, displays much less homoplasy than the first and, thus, is easier searchable. Consequently, less effort is needed here to arrive at a similar result. The 385 taxa dataset is comparable to the 89 taxa set in terms of homoplasy but is much larger. Unsurprisingly, the Bremer support settles later here. The same reasoning applies to the largest, 567 taxa dataset, for which the 90%-level is reached only after 17 iterations. In view of the range of different taxon and character sampling covered by these four analyses, saturation appears to happen at quite similar times. Obviously, the 90%-level is an arbitrary measure, and one might want to extend analyses until an average 95% of the final Bremer support differences are reached. This, however, would not change much the relative times at which that is achieved. As a rule of thumb it appears that not much is gained relative to the additional effort when searches are extended beyond 20 iterations, at least not for datasets that fall within the size range observable in current publications. For datasets with a still smaller taxon sampling and still less homoplasy, for which using the ratchet principally does not save time, a single simple addition search (saving a limited number of trees) may already yield Bremer support values close enough to the "true" values (found, e.g, by branch-and-bound searches). As soon as the size and structure of a dataset entails that shortest trees are principally only encountered when at least a random addition search is performed, using the ratchet and the 90%-levels found here as a guideline seems a reasonable strategy.

## Conclusion

One consequence of the above findings is that calculating bootstrap- or jackknife proportions with narrow confidence intervals can be achieved with less expense than often thought, even for very large datasets. In turn, this means that iterated bootstrap methods that aim at reducing statistical bias inherent to bootstrap proportions are more feasible, since the individual bootstrap searches may be performed using less time-intensive heuristic searches during each replicate.

As a further consequence of these reflections, finding bootstrap or jackknife proportions with reasonable confidence can be achieved with much less costs than trying to find the correct Bremer support for large datasets. Bootstrap or jackknife values saturate immediately after one simple addition search, while finding satisfyingly accurate Bremer support may require 20 or more iterations, much depending on what one subjectively accepts as "satisfyingly accurate" (90% of the maximum difference that could be achieved with exhaustive searches ?). If we take the 385 taxa dataset as an example, bootstrap support with 1% accurateness at ≥95% could be gained by 1825 simple searches (with TBR), while Bremer support would require N*(1+13*2) such searches, where *N *is the number of nodes to test (typically far less than the theoretically possible number of internal nodes). If we approximate that TBR swapping on one tree takes roughly equally long irrespective of the particular resampled matrix or the particular constraints in effect at a given node, calculating Bremer support for the 339 nodes resolved in the strict consensus [48] takes five times longer than bootstrapping or jackknifing (6.5 times if 95% of the final support difference are to be achieved, which on average happens at the 17^th ^iteration for that dataset).

The relative speed of both methods strongly varies with the accurateness aimed at, but even calculating 19592 jackknife iterations needed for 0.5% accurateness at nodes with ≥85% (meaning that the size of the confidence interval matches the precision at which BS/JS proportions usually are reported) is faster than obtaining Bremer support with the precision outlined above, which may only be the case beyond an equivalent of 339*(1+(50*2)) = 34239 such jackknife iterations. The relatively higher performance of jackknifing and bootstrapping further increases with the taxon sampling size. Strictly speaking, a comparison of the speed at which both support types are computable makes not much sense, due to the fundamental differences of both. On the other hand, along with considerations on the interpretability of Bremer support compared to the bootstrap and jackknife [41], such practical considerations may assist in choosing which support type to report when the time available for analyses is limited.

## Methods

Four different datasets were chosen that differed not only in the number of taxa sampled but also in the kind of DNA sequence data used (e.g., absolute number of informative characters, overall sequence divergence, overall support levels and levels of homoplasy). Two are comparatively large: a three-gene dataset [44], containing DNA sequences of 18S rDNA, *rbc*L, and *atp*B (4592 characters, 2153 parsimony informative) for 567 angiosperm taxa, and a *matK *dataset [48] for 385 angiosperm taxa (1749 chars, 1075 parsimony informative). Two are of intermediate size: one, using the *trnK *intron [49], provides high overall support (89 taxa, 3538 characters, 1441 parsimony informative), the other [50] less so (*trnL-F *and *rps4*, 86 taxa, 1204 characters, 286 parsimony informative).

### Bootstrap and jackknife

The fast search approach (no branch swapping) has been frequently shown to yield considerably lower support values [12-15]. The same applies to less thorough branch swapping algorithms such as NNI (as opposed to TBR; [12,15]. Also, RAS has no beneficial effects on BS/JS as compared to the simple addition sequence – a conclusion based on a 173 taxa set [15]. For still larger datasets, RAS has been frequently shown to be inferior to other strategies such as the parsimony ratchet. In consequence, an extensive testing of the effect of RAS on the datasets analyzed here appeared not warranted. Therefore, the effect of using a limited number of ratchet iterations per jackknife resampling replicate was assessed with help of the author's short C++ program PRAT [24] in conjunction with PAUP* [19].

Since too few replications would hamper contrasting the strategies due to rather wide confidence intervals around the JS values, and since a series of repeated ratchet analyses soon becomes quite time consuming, 500 replicates served as a compromise between variance and computation times. Since the sign test used (see below) ignores the magnitude of differences and since stochastic deviations from the expected BS/JS equally likely are positive or negative, the accurateness achieved with 500 replications should be sufficient in view of the high number of nodes contrasted. Note, however, that knowing the BS or JS of a particular clade with relatively high confidence would require more iterations for values ≥90% (Fig. 1). For the two largest datasets, the ratchet found much shorter trees within the first 10–20 ratchet iterations than simple searches or RAS in PAUP* (in line with common observations; see also Table 1 in [43]). Thus, in terms of the proximity to the putatively minimal tree score, the contrast between TBR on one shortest tree and 20 ratchet iterations is higher than that between 20 ratchet iterations and any number of additional iterations. Therefore, it was first tested whether searches of 10 and 20 ratchet iterations per BS/JS replicate make a difference prior to using more iterations.

To compare jackknife values on two trees, the JS values of all nodes resolved in one tree were compared to the corresponding JS values of the other tree using (a) a sign test and (b) a Wilcoxon test. The first excludes the magnitude of the difference between a pair and only takes pairs with differing values into consideration. The second was used to crosscheck the outcome of the sign test by incorporating information on the magnitude of divergent pairs and retaining information on how many pairs actually consist of equal values. Both tests fully agreed on rejecting or accepting an overall equality of JS (see Table 1).

### Bremer support

For each of the four datasets, 25 nodes of the strict consensus tree were randomly chosen (using JAVA's Random class) and subjected to a simple heuristic search in PAUP*, saving only one tree, followed by a parsimony ratchet analysis of 50 iterations (25% characters with double weight, saving one tree). Thus, unlike in the jackknife analyses, not all resolved nodes were monitored due to the considerable search time needed per node. All analyses were performed with PRAP [43] in combination with PAUP*. Monitoring and evaluating the change of support over time was achieved with additional small JAVA classes written for this study.
